# Effects of *Fusarium graminearum* and *Fusarium verticillioides* Infection on Sweet Corn Quality During Postharvest Storage

**DOI:** 10.3390/foods14234147

**Published:** 2025-12-03

**Authors:** Yihan Xue, Shaoyue Liu, Qianzi Nie, Xinru Zhang, Yan Zhao, Yanfei Li, Haoxin Lv

**Affiliations:** 1 Food Engineering Technology Research Center, Key Laboratory of Henan Province, Henan University of Technology, Zhengzhou 450001, China; 2 School of Food and Strategic Reserves, Henan University of Technology, 100 Lianhua Street, Gaoxin District, Zhengzhou 450001, China

**Keywords:** sweet corn, postharvest storage, *Fusarium graminearum*, *Fusarium verticillioides*, fungal infection

## Abstract

Sweet corn is highly susceptible to infection by *Fusarium graminearum* (*F. graminearum*) and *Fusarium verticillioides* (*F. verticillioides*) during storage, which substantially compromises its nutritional quality and economic value. However, the specific effects of *F. graminearum* and *F. verticillioides* on sweet corn quality during postharvest storage remain poorly understood. This study systematically explored the effects of *F. graminearum* and *F. verticillioides* infection on sweet corn quality by evaluating the changes in color, hardness, weight loss rate, malondialdehyde (MDA) content, surface fungal spore count, soluble protein content, and soluble sugar content. Results indicated that the critical time points for visible deterioration were 24 h post-inoculation for *F. graminearum* and 36 h for *F. verticillioides*. Compared with the control group, both infections caused significant darkening of kernel color and a marked increase in surface fungal spore counts. Notably, *F. verticillioides* infection was associated with a significant increase in MDA content, indicating enhanced oxidative stress in infected kernels. These findings elucidate the mechanisms of pathogen-induced quality degradation in sweet corn and provide a scientific basis for preserving and enhancing the value of agricultural products and ensuring food safety.

## 1. Introduction

Sweet corn (*Zea mays* L. *saccharata* Sturt), also known as fresh corn or glutinous corn, refers to young corn varieties harvested at the milk stage. It is widely appreciated by consumers due to its thin pericarp, abundant juice, crisp and tender texture, and high nutritional value [1]. Moreover, its fruit-like characteristics render it suitable for both fresh consumption and culinary use. Sweet corn has become a globally important economic crop, primarily cultivated in the United States, France, South Korea, and the northeast regions of China. It is reported that China has surpassed the United States in sweet corn production, with a planting area of 5.3 million hectares, making itself as the world’s largest producer and consumer. The country has developed a comprehensive planting and processing system, playing a crucial role in ensuring food security and promoting agricultural economic development [2].

Sweet corn is derived from the mutation of the genes involved in common corn starch synthesis metabolism, which has a unique flavor of both fruits and grains, rich in nutrients [3,4]. Due to the high moisture and soluble sugar content, sweet corn is highly susceptible to spoilage during storage [5]. When stored under ambient conditions, sweet corn remains fresh for only 2–3 days [6]. Moreover, its shelf life is inherently short, limited primarily to the milk ripening stage, which lasts approximately 3 to 7 days [7,8]. During this stage, the moisture content and levels of soluble sugars, such as sucrose and glucose, reach their maximum. However, postharvest physiological metabolism in sweet corn is highly active, with respiratory intensity significantly exceeding that of field corn, resulting in rapid degradation of key nutritional components, including vitamin C and lutein [9]. Additionally, the high moisture and sugar content render sweet corn particularly vulnerable to various pathogenic microorganisms during storage, leading to mold growth and spoilage [5]. Pathogen-infected sweet corn not only suffers substantial losses in quality and yield [10], but may also produce mycotoxins during fungal proliferation, which can induce acute or chronic toxicity in humans and animals [11], thereby posing a significant risk to public health. These challenges severely restrict the postharvest circulation and marketability of sweet corn. Therefore, developing effective strategies to suppress the onset and spread of postharvest diseases through optimized prevention and control measures has become a critical priority in contemporary agricultural and food science research.

Maize ear rot is one of the primary fungal diseases affecting the late growth stage and postharvest storage of corn, and it is a prevalent disease in maize-producing regions worldwide, resulting in substantial yield losses and significant deterioration of corn quality [12]. The disease is caused by infection with various pathogens, either individually or synergistically [13]. Global studies have consistently identified *Fusarium verticillioides* (*F. verticillioides*) and *Fusarium graminearum* (*F. graminearum*) as the major pathogenic species [14,15,16,17,18,19]. These filamentous fungi can proliferate rapidly under conditions of 25–35 °C and relative humidity exceeding 85%. Furthermore, they are capable of persistent postharvest infection through mechanical wounds or latent mycelial structures, leading to the development of water-soaked lesions on kernels and the formation of mold layers. This results in marked deterioration of sensory quality, including textural softening and other undesirable changes [20].

More seriously, *F. graminearum* and *F. verticillioides* produce harmful secondary metabolites, including the mycotoxins deoxynivalenol (DON), zearalenone, and fumonisins (FB1) [21,22]. These toxins contribute to the accelerated deterioration of grain quality, such as impaired starch gelatinization properties and disrupted sugar metabolism. Of particular concern, consumption of food contaminated with zearalenone by pregnant animals, including humans, may result in miscarriage, stillbirth, or fetal abnormalities [23]. Moreover, excessive intake of zearalenone can lead to mycotoxicosis, commonly referred to as “fungal poisoning”, posing a significant health risk to both animals and humans [24].

This study investigated and documented the infection process of *F. graminearum* and *F. verticillioides* in sweet corn under normal storage conditions at 25 °C, aiming to identify the critical time points of infection. By evaluating color, hardness, weight loss rate, malondialdehyde (MDA) content, surface fungal spore count, soluble protein content and soluble sugar content, the changes in sweet corn quality before and after infection were systematically analyzed. This research aims to establish a theoretical foundation for understanding pathogen-induced quality degradation in sweet corn and to develop strategies for controlling and preventing postharvest quality deterioration prior to the critical infection stages of *F. graminearum* and *F. verticillioides*.

## 2. Materials and Methods

### 2.1. Materials

*F. graminearum* CFCC7875 was acquired from the China General Microbiological Culture Collection Center, and *F. verticillioides* 1DMA was maintained at School of Food and Strategic Reserves, Henan University of Technology (34°47′2″ N, 113°32′39″ E), Zhengzhou City, Henan Province, China. Fresh sweet corn samples of Zhengbainuo No. 5 were obtained from local farmers in Xingyang County (34°47′25″ N, 113°21′16″ E), Zhengzhou City, Henan Province, China.

### 2.2. Preparation of Pathogen Spore Suspension

Based on the method described in previous studies [15,25,26], *F. graminearum* and *F. verticillioides* strains were inoculated onto potato dextrose agar (PDA) plates and incubated at 28 °C for 7 days. Subsequently, the activated mycelia were transferred to carboxymethyl cellulose (CMC) agar plates and incubated under the same conditions to induce sporulation. Sporulated mycelial fragments were then transferred to 250 mL conical flasks containing 100 mL of CMC liquid medium and cultivated at 28 °C with shaking at 180 rpm for 7 days. Following filtration through sterile gauze to remove mycelial debris, the spore suspension concentration was determined microscopically using a hemocytometer. The spore suspension was adjusted to a final concentration of 1 × 10^6^ CFU/mL and was stored at 4 °C for future use.

### 2.3. Postharvest Treatment and Sampling of Sweet Corn

According to the method described by Liu et al. [27], sweet corn cobs were divided into three groups: the control (CK) group, the *F. graminearum* infection (FG) group and the *F. verticillioides* infection (FV) group. In brief, 20 µL of sterile water (CK group), *F. graminarum* spore suspension (FG group) or *F. verticillioides* spore suspension (FV group), each adjusted to a final concentration of 1 × 10^6^ CFU/mL, was injected into the equatorial region of individual sweet corn cobs. The inoculated samples were then incubated under controlled conditions at 25 °C and 75–85% relative humidity for 60 h [4]. Sampling time points were established as follows: for the CK group, samples were collected at 0, 8, 12, 16, 24, 36, 48, and 60 h; for the FG group, at 0, 8, 16, 24, 36, 48, and 60 h; and for the FV group, at 0, 12, 24, 36, 48, and 60 h. At each time point, the following parameters were recorded: visual assessment, color, hardness, weight loss rate, MDA content, surface fungal spore count, soluble protein content and soluble sugar content. Whole sweet corn cobs were used for visual assessment and weight loss rate measurement, whereas sweet corn kernels were utilized for the evaluation of other quality parameters.

### 2.4. Visual Assessment of Sweet Corn

Based on the method described by Wu et al. [28] with minor modifications, the phenotypic characteristics—including color, morphology, and structure integrity—of sweet corn samples were visually evaluated under standardized conditions using unaided vision. During observation, a Canon EOS R6 camera (Canon Inc., Tokyo, Japan) was used to capture phenotypic data with a fixed focal length and consistent imaging parameters. Original images were saved in JPG format for subsequent visual assessment and documentation.

### 2.5. Color Measurement of Sweet Corn

The color was measured using a CR-410 colorimeter manufactured by Kefei (Hangzhou, China) Instrument Co., Ltd. following the method described by Zhang et al. [5]. The instrument was preheated for 3 min to stabilize the optical system, after which white board calibration was performed and standard measurement settings were configured. Once the system reached stability, the L* and b* color space parameters were recorded for the sweet corn kernel samples.

### 2.6. Hardness Measurement of Sweet Corn

The hardness was measured following the method described by Liu et al. [29]. A TA-XT Plus texture analyzer manufactured by Stable Micro Systems (Godalming, UK) was used for the measurement. Prior to the experiment, the instrument was powered on, and the Texture Expert 32 software system was initiated. A P/36R cylindrical probe (diameter: 36 mm) was attached for testing. The hardness measurement parameters were set according to Table S1, and data were recorded in real time using the Texture Expert 32 software.

### 2.7. Measurement of Weight Loss Rate in Sweet Corn

The weight loss rate was measured according to the method of Liu et al. [29]. The weight was measured using an analytical balance. The initial weight was recorded immediately, after which the samples were promptly returned to the incubator for subsequent weighing. The weight loss percentage was calculated by comparing the weight at each sampling time point with the initial weight. The difference was divided by the initial weight, multiplied by 100, and expressed as the weight loss percentage (*w*/*w*, %).

### 2.8. Measurement of MDA Content in Sweet Corn

The MDA content was quantified using spectrophotometry based on the method described by Zhang et al. [30]. Experimental samples included the FG and FV groups, along with the CK group, all of which were stored at −80 °C. The MDA reagent kit (Shanghai Enlink Biotechnology Co., Ltd., Shanghai, China) was removed from 4 °C storage and allowed to equilibrate to room temperature prior to use, following the manufacturer’s instructions.

### 2.9. Assessment of Surface Fungal Spore Count in Sweet Corn

The surface fungal spore count of sweet corn was determined according to the method described by Wang et al. [31]. Spore count was observed and recorded using a hemocytometer (Merck KGaA, Darmstadt, Germany).

### 2.10. Measurement of Soluble Protein Content in Sweet Corn

The soluble protein content was quantified using spectrophotometry based on the method described by Cheng et al. [32]. Samples from the FG and FV experimental groups, along with the CK group, were selected for analysis. All samples had been stored at −80 °C. The soluble protein assay kit (Shanghai Enlink Biotechnology Co., Ltd., Shanghai, China) was removed from 4 °C storage, allowed to equilibrate to room temperature, and then used in accordance with the manufacturer’s instructions.

### 2.11. Measurement of Soluble Sugar Content in Sweet Corn

The soluble sugar content was quantified using spectrophotometry according to the method described by Wang et al. [33]. Samples from the FG and FV experimental groups, as well as the CK group, were selected for analysis. All samples had been stored at −80 °C. The soluble sugar assay kit (Shanghai Enlink Biotechnology Co., Ltd., Shanghai, China) was removed from 4 °C storage, allowed to equilibrate to room temperature, and then used in accordance with the manufacturer’s instructions.

### 2.12. Data Statistical Analysis and Processing Methods

Experimental data were processed and organized using Microsoft Excel 2019, and quantitative analysis results were visualized using OriginLab 2018. Statistical validation was performed using IBM SPSS Statistics 27 to assess significant differences among groups via one-way analysis of variance (ANOVA). Unless otherwise stated, all measured parameters were based on three biological replicates.

## 3. Results

### 3.1. Visual Observation of Sweet Corn

**Figure 1:**
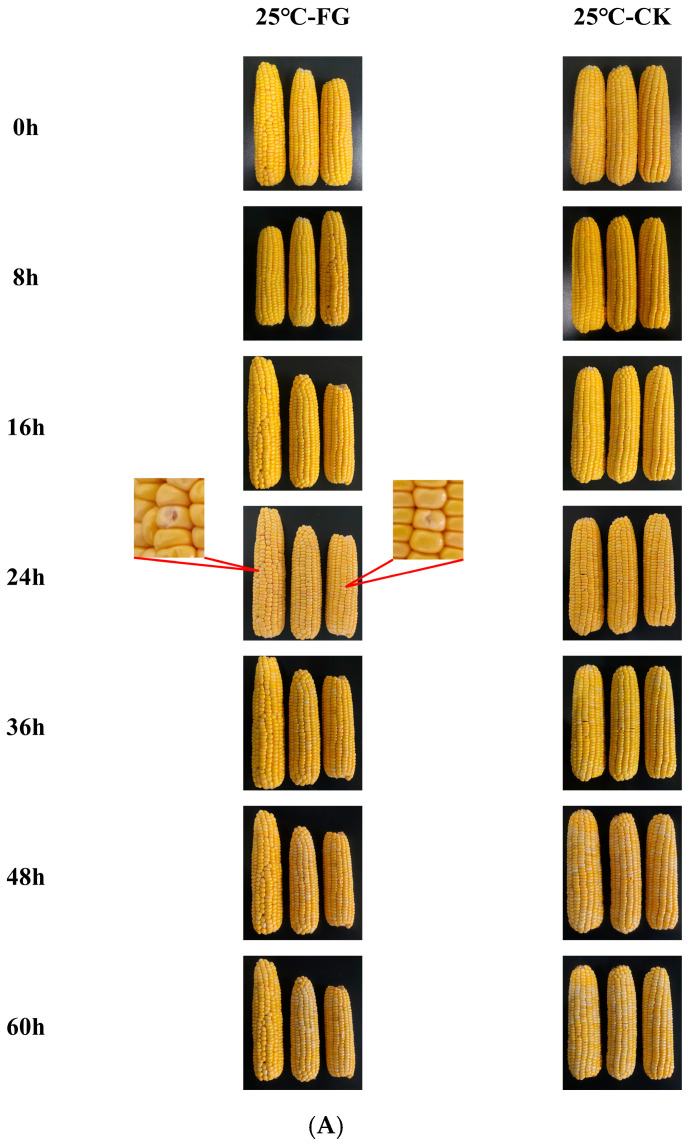

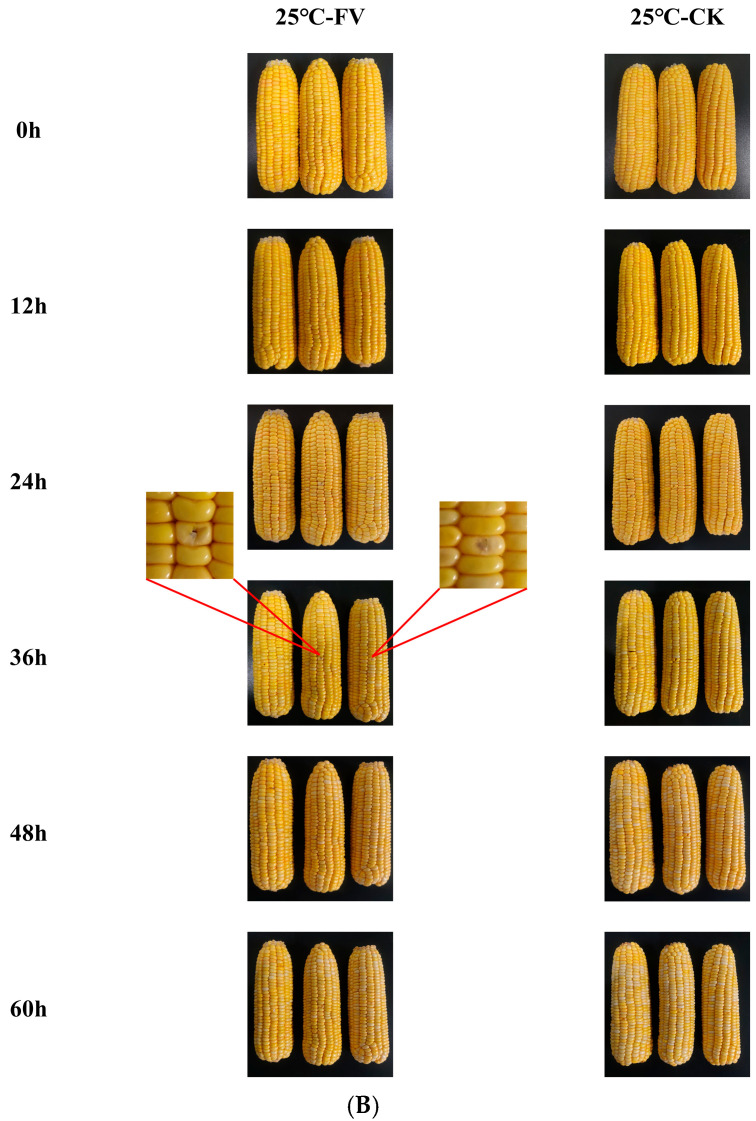
Effects of *F. graminearum* (**A**) and *F. verticillioides* (**B**) infection on sweet corn appearance.

The visual changes in sweet corn are presented in Figure 1. As storage duration increased, both *F. graminearum* and *F. verticillioides* exhibited significant mycelial proliferation on the sweet corn ears, confirming their ecological adaptability to the nutritional substrates associated with the *Fusarium* genus. At the initial stage of storage (0–24 h), sweet corn samples from the FG and FV groups as well as the CK group maintained good color integrity, with no visible signs of microbial contamination. By 24 h of storage, white mycelia became apparent on the surface of samples in the FG group (Figure 1A), indicating that *F. graminearum* had initiated surface colonization; in contrast, the FV and the CK groups showed no significant changes at this time (Figure 1B). Mycelial growth in the FG group further intensified compared to the 24 h observation (Figure 1A). At 36 h of storage, white mycelia were observed on the surface of sweet corn in the FV group (Figure 1B), suggesting active infection by *F. verticillioides*. These observations indicate that fungal colony development progressively increased with prolonged storage, reflecting a corresponding increase in mold severity. As storage time extended further, mycelial proliferation in both FG and FV groups became markedly more extensive, with mycelial density showing a positive correlation with storage duration. The kernel color gradually changed from bright yellow to dull yellowish-brown. In contrast, the CK group exhibited neither mycelial growth nor noticeable color alteration throughout the experiment period. Therefore, under the storage condition of 25 °C, the critical time point for *F. graminearum* infection is 24 h, while that for *F. verticillioides* infection is 36 h.

### 3.2. Color Variation in Sweet Corn

Color changes in sweet corn during storage are presented in Table 1 and Table 2. According to previous studies [34,35], the L* value represents lightness, with higher values indicating greater kernel brightness; the b* value reflects the yellow-blue chromatic component, where higher values correspond to a more intense yellow hue. As shown in Table 1, over the storage period, the L* value in the FG group exhibited a relatively stable trend but was significantly lower than that of the CK group at the critical infection time point of 24 h. The b* value initially decreased and then increased, being significantly lower than the CK group at 24 h and significantly higher at 60 h. As shown in Table 2, with prolonged storage, the L* value in the FV group showed minimal overall change, although it was significantly lower than that of the CK group at the critical infection time point of 36 h. The b* value displayed a trend of initial increase followed by decrease. These results indicate that, compared to the CK group, infection with *F. graminearum* and *F. verticillioides* significantly darkened the color of sweet corn kernels. It has been reported that the pathogen can invade the kernels and ears, leading to ear rot, which results in the formation of grayish-white or pale red mold colonies on the kernels, characterized by white, fluffy or woolly mycelial growth [36]. Consequently, these fungal colonies may alter the appearance of sweet corn kernels by darkening their color.

### 3.3. Hardness Change of Sweet Corn

**Figure 2:**
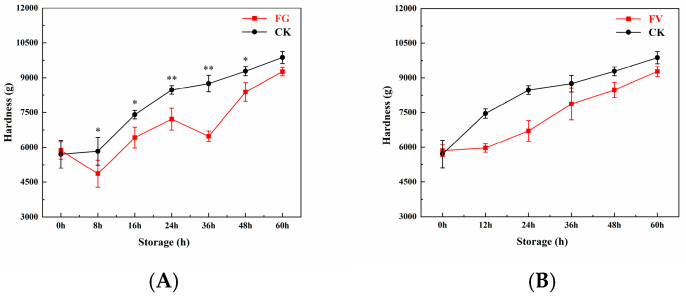
Changes in hardness of sweet corn during *F. graminearum* (**A**) and *F. verticillioides* (**B**) infection. Note: asterisks (*) denote significant differences over time within each treatment between sweet corn kernels (*p* < 0.05). Asterisks (**) denote extremely significant differences over time within each treatment between sweet corn kernels (*p* < 0.01).

Hardness changes in sweet corn during storage are shown in Figure 2. The results indicate that, as storage duration increased, the hardness of sweet corn in the FG and FV groups as well as the CK group exhibited an overall increasing trend. Except at 60 h, the hardness in the FG group was significantly lower than that of the CK group (Figure 2A), whereas no significant differences were observed between the FV group and the CK group throughout the storage period (Figure 2B). In summary, during infection of sweet corn by *F. graminearum* and *F. verticillioides*, sweet corn gradually became harder with prolonged storage time.

### 3.4. Changes in Weight Loss Rate of Sweet Corn

**Figure 3:**
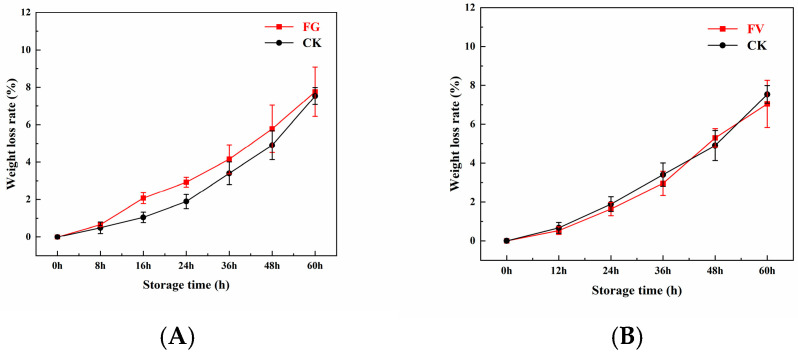
Changes in weight loss rate of sweet corn during *F. graminearum* (**A**) and *F. verticillioides* (**B**) infection.

The dynamic changes in the weight loss rate of sweet corn are displayed in Figure 3. During storage, the weight loss rates of the FG and FV groups as well as the CK group exhibited an increasing trend. However, no significant differences were observed between the FG group and the CK group (Figure 3A) or between the FV group and the CK group (Figure 3B).

### 3.5. Changes in MDA Content of Sweet Corn

**Figure 4:**
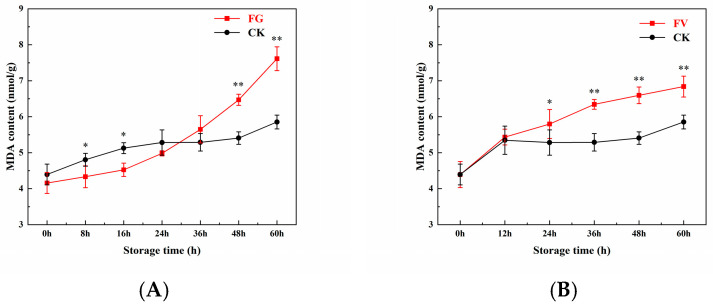
Changes in malondialdehyde (MDA) content of sweet corn during *F. graminearum* (**A**) and *F. verticillioides* (**B**) infection. Note: asterisks (*) denote significant differences over time within each treatment between sweet corn kernels (*p* < 0.05). Asterisks (**) denote extremely significant differences over time within each treatment between sweet corn kernels (*p* < 0.01).

As shown in Figure 4, with prolonged storage time, the MDA content in both the FG and FV groups increased compared to the CK group. The rate of increase in MDA content in the CK group was significantly lower than that in the FG and FV groups. The initial MDA content in the sweet corn samples was 4.16 nmol/g in the FG group (Figure 4A), 4.39 nmol/g in the FV group (Figure 4B), and 4.39 nmol/g in the CK group, with no significant differences among them. At 36 h of storage, the MDA content in the FV group was significantly higher than that of the CK group (Figure 4B). In summary, during infection of sweet corn by *F. graminearum* and *F. verticillioides*, MDA content increased with extended storage time; compared to the CK group, infection with *F. verticillioides* led to a significant elevation in MDA content.

### 3.6. Changes in Surface Fungal Spore Count of Sweet Corn

**Figure 5:**
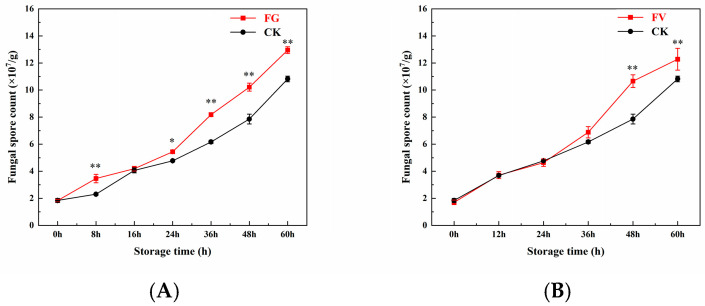
Changes in fungal spore count of sweet corn during *F. graminearum* (**A**) and *F. verticillioides* (**B**) infection. Note: asterisks (*) denote significant differences over time within each treatment between sweet corn kernels (*p* < 0.05). Asterisks (**) denote extremely significant differences over time within each treatment between sweet corn kernels (*p* < 0.01).

As shown in Figure 5, for the FG and FV groups, the surface fungal spore count of sweet corn gradually increased with prolonged storage time. The rate of increase in fungal spore count was significantly higher in the FG and FV groups than that in the CK group. No significant differences were observed among the FG group, the FV group, and the initial samples of the CK group. However, at 48–60 h of storage, the fungal spore count in the FG group was significantly elevated compared to the CK group (Figure 5A), suggesting that *F. graminearum* infection promoted spore proliferation. Similarly, at 24–60 h, the fungal spore count in the FV group was significantly higher than that in the CK group (Figure 5B), indicating that *F. verticillioides* infection also enhanced spore accumulation. Overall, during sweet corn infection by *F. graminearum* and *F. verticillioides*, the surface fungal spore count increased significantly over time; compared to the CK group, infection with either pathogen led to a marked increase in surface fungal spore load.

### 3.7. Changes in Soluble Protein Content of Sweet Corn

**Figure 6:**
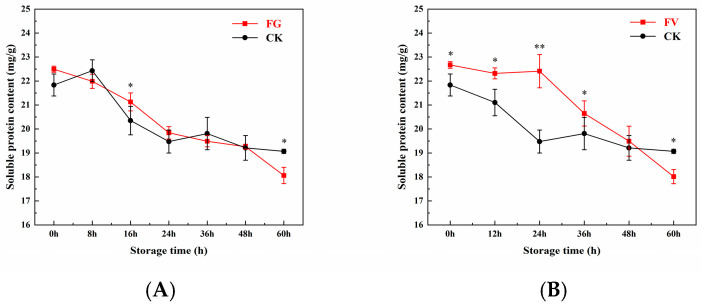
Changes in soluble protein content of sweet corn during *F. graminearum* (**A**) and *F. verticillioides* (**B**) infection. Note: asterisks (*) denote significant differences over time within each treatment between sweet corn kernels (*p* < 0.05). Asterisks (**) denote extremely significant differences over time within each treatment between sweet corn kernels (*p* < 0.01).

As shown in Figure 6, the soluble protein content in both the FG and FV groups as well as the CK group exhibited a decreasing trend over the storage period. The rate of protein degradation in the CK group was significantly lower than that observed in the FG and FV groups. At the beginning of storage, the soluble protein contents in the FG, FV, and CK groups were 22.49, 22.67, and 21.83 mg/g, respectively; after 60 h of storage, these values decreased to 18.06, 18.02, and 19.07 mg/g, respectively. The final protein levels in the FG and FV groups were significantly lower than that of the CK group by 9.2% and 9.4%, respectively (*p* < 0.05). These results indicate that during sweet corn infection by *F. graminearum* and *F. verticillioides*, the soluble protein content declines significantly with prolonged storage time.

### 3.8. Changes in Soluble Sugar Content of Sweet Corn

**Figure 7:**
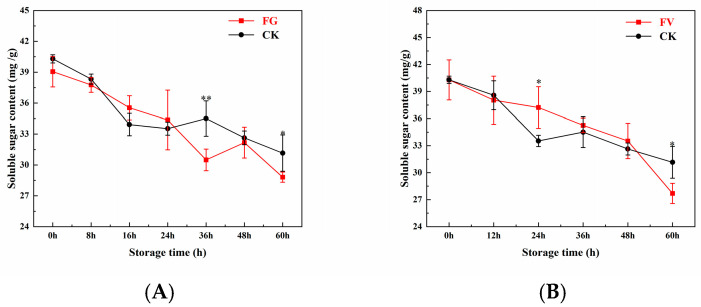
Changes in soluble sugar content of sweet corn during *F. graminearum* (**A**) and *F. verticillioides* (**B**) infection. Note: asterisks (*) denote significant differences over time within each treatment between sweet corn kernels (*p* < 0.05). Asterisks (**) denote extremely significant differences over time within each treatment between sweet corn kernels (*p* < 0.01).

As shown in Figure 7, the soluble sugar content in both the FG and FV groups decreased progressively over time compared to the CK group. The rate of soluble sugar degradation in the CK group was significantly lower than that observed in the FG and FV groups. At the beginning of storage, the soluble sugar content in the FG, FV, and CK groups was 39.05, 39.05, and 40.30 mg/g, respectively, with no significant differences among them. After 60 h of storage, these values declined to 28.81, 27.70, and 31.16 mg/g, respectively. The final soluble sugar levels in the FG and FV groups were significantly lower than that of the CK group by 7.5% and 7.5%, respectively (*p* < 0.05). In summary, during the infection of sweet corn by *F. graminearum* and *F. verticillioides*, the soluble sugar content decreased significantly with prolonged storage time.

## 4. Discussion

Sweet corn is rich in soluble sugars and various nutrients, and is highly favored by consumers due to its unique taste and pleasant flavor. Among the various quality indicators, color represents a key product attribute, as it provides an intuitive indication of sweet corn freshness [37]. The L* and b* values are commonly used to assess the freshness of sweet corn kernels [5,38]. In this study, during storage, the initially uninfected corn kernels appeared plump and exhibited bright, vivid yellow coloration (Figure 1 and Table 1). The observed trends in brightness and yellowness of sweet corn align with those reported by [39], showing consistency over increasing storage duration. Meanwhile, as storage time extended, the degree of white mycelial growth on the surface of sweet corn in the FG and FV groups increased significantly, with mycelial density showing a positive correlation with storage time. Last but not the least, this study identified the critical infection time points, 24 h for *F. graminearum* and 36 h for *F. verticillioides*, which could be leveraged to implement timely interventions for controlling and preventing infections caused by these pathogens before the onset of critical infection stages.

During the infection of sweet corn by *F. graminearum* and *F. verticillioides*, kernel hardness gradually increased with prolonged storage time (Figure 2). However, the hardness in the FG and FV groups was lower than that in the CK group. It has been reported that sweet corn hardens during storage due to water loss and increased cell membrane permeability [40]. Research indicates that *F. graminearum* produces higher levels of polygalacturonase and cellulase enzyme activities compared to other pathogens. These enzymes efficiently degrade pectin and cellulose, thereby weakening intercellular adhesion and leading to a looser grain structure. Moreover, the activity of cell wall-degrading enzymes in sweet corn is positively correlated with the reduction in kernel hardness. Especially in the late stages of infection, sustained cellulase activity further accelerates cell wall disintegration, resulting in decreased textural firmness. In addition, previous research has shown that corn kernels infected by *F. graminearum* exhibit a 30–50% reduction in hardness within seven days post-infection, which is significantly greater than the 20–35% reduction observed in kernels infected by *F. verticillioides* [41].

*F. graminearum* and *F. verticillioides* are two common pathogenic fungi affecting maize, capable of significantly influencing the weight loss rate during sweet corn storage. In this study, infection of sweet corn by *F. graminearum* and *F. verticillioides* resulted in an increasing weight loss rate over time (Figure 3). It has been reported that in ears infected by *F. graminearum*, the husk adheres tightly to the kernels, restricting water loss from the exterior, while structural damage within the kernels leads to rapid localized water evaporation, creating an imbalanced state of “internal drying—external retention”, ultimately contributing to an overall increase in weight loss. Mycotoxins produced by *F. graminearum*, such as DON, can inhibit maize respiration and indirectly accelerate water loss [42]. Studies have shown that after 21 days of storage, the weight loss rate of sweet corn infected with *F. graminearum* is 15–20% higher than that of the uninfected group, a difference directly associated with fungal-induced tissue damage and enhanced water evaporation [43]. Fumonisins produced by *F. verticillioides*, such as FB1, may impair mitochondrial function, thereby exacerbating disruptions in energy metabolism and reducing dry matter accumulation in the kernels. In addition, pathogenic fungi directly consume kernel nutrients, including starch and protein, further contributing to weight loss [44]. Therefore, it is hypothesized that these two fungi promote water loss and tissue degradation through distinct but complementary mechanisms. Concurrently, cell wall degradation and cell structural disruption in sweet corn increase kernel surface porosity, accelerating the rate of water evaporation.

The lipid peroxidation level in sweet corn was assessed by measuring MDA content [45]. In this study, MDA levels in the infected group increased progressively with prolonged storage time (Figure 4). Compared to the CK group, infection with *F. verticillioides* significantly elevated MDA content in sweet corn. This increase may be associated with a reduction in bound water content, as a previous study has reported a correlation between bound water levels and lipid oxidation [46]. Furthermore, it has been shown that *F. verticillioides* forms infection structures resembling appressoria, characterized by enlarged hyphal tips, which can influence the peroxidation status of corn tissues. These findings suggest that *F. verticillioides* infection induces oxidative stress in sweet corn, likely contributing to decreased bound water content.

Fresh sweet corn contains high levels of sugar and moisture, making it highly susceptible to infection by pathogenic molds, which can lead to rapid quality deterioration [43]. This study found that during the infection by *F. graminearum* and *F. verticillioides*, the number of fungal spores on the surface of sweet corn increased significantly with prolonged storage time (Figure 5). Compared to the CK group, infection with *F. graminearum* and *F. verticillioides* markedly elevated surface fungal spore counts. This increase may be attributed to the rise in the free water content resulting from the conversion of bound water within the kernels [31].

Soluble protein is a key nutritional component in sweet corn. In this study, during infection by *F. graminearum* and *F. verticillioides*, the soluble protein content in sweet corn decreased significantly with prolonged storage time (Figure 6). This decline may be attributed to the degradation or structural damage of proteins within the kernels. It has been reported that *F. graminearum* can directly degrade corn storage proteins by secreting serine proteases (e.g., FgPr1) and metalloproteases (e.g., FgMep1), while simultaneously activating the host ubiquitin-proteasome system (UPS) to accelerate the clearance of damaged proteins [47]. Meanwhile, DON inhibits ribosomal function, thereby interfering with protein synthesis and leading to a 23% reduction in total soluble protein levels within 14 days [48]. Another contributing factor may be mycotoxin-induced cell membrane destruction. FB1 produced by *F. verticillioides* inhibits ceramide synthase, disrupting membrane lipid metabolism and indirectly promoting protein degradation. By the 10th day of storage, soluble protein content had declined to 65% of its initial value, accompanied a 50% decrease in the activity of membrane-bound enzymes such as H^+^-ATPase [49].

Soluble sugar is a key indicator of sweet corn taste quality and directly determines its sweetness [50]. In this study, infection with *F. graminearum* and *F. verticillioides* led to a significant decrease in soluble sugar content in sweet corn (Figure 7). It has been reported that α-amylase (FgAmy1) secreted by *F. graminearum* reaches peak activity three days post-infection, catalyzing the breakdown of starch into glucose and maltose. However, DON inhibits plant sugar transporters such as SUT1, resulting in a rapid decline in sugar levels [51]. In addition, FB1 increases the consumption rate of soluble sugars by interfering with the host carbohydrate metabolism signaling pathways, including the inhibition of hexokinase activity. This effect is further exacerbated under high-temperature conditions (25–30 °C) [52]. Studies have also shown that *F. verticillioides* actively absorbs host-derived sugars via the high-affinity glucose transporter HGT1, contributing to a marked reduction in soluble sugar content following infection [44].

## 5. Conclusions

Sweet corn is a variety of super-sweet maize. During the milk-ripe stage, its kernels are tender and rich in water-soluble polysaccharides, starch, and lipids, making it highly favored by consumers. However, it is susceptible to invasion by pathogenic microorganisms during postharvest storage. This study investigated quality changes in sweet corn kernels and identified key infection time points following exposure to *F. graminearum* and *F. verticillioides*. Results showed that under storage condition of 25 °C, the critical infection time point for the FG group was 24 h, while that for the FV group was 36 h. With prolonged storage time, sweet corn kernels gradually became firmer and darker in color. The weight loss rate and MDA content increased, surface fungal spore counts rose, whereas soluble protein and soluble sugar contents declined. Compared with the CK group, infection by the FG and FV groups resulted in significant darkening of kernel color and a marked increase in surface fungal spore count. Infection by the FV group also led to a significant elevation in MDA content. These findings indicate that the *F. graminearum* and *F. verticillioides* infections can accelerate the deterioration of sweet corn kernel quality during postharvest storage. This study elucidates the mechanisms underlying pathogen-induced quality degradation in sweet corn and identifies a critical time window for effective control and prevention of postharvest quality deterioration prior to the onset of critical infection stages caused by *F. graminearum* and *F. verticillioides*, thereby providing actionable guidance for farmers and storage facilities to implement timely interventions to mitigate fungal contamination. In addition, this study was conducted under specific storage conditions of 25 °C and 75–85% relative humidity and focused specifically on infections caused by *F. graminearum* and *F. verticillioides*. Future research should extend to other storage environments, additional fungal pathogens, and the evaluation of antifungal treatment efficacy.

## Figures and Tables

**Table 1:** Color changes in sweet corn during *F. graminearum* infection.

Color	Storage Time	CK	FG
L*	0 h	69.80 ± 0.72 a	69.42 ± 0.48 ab
	8 h	69.78 ± 0.70 a	68.76 ± 1.01 ab
	16 h	69.53 ± 0.93 ab	69.78 ± 0.35 a
	24 h	70.32 ± 0.16 a	66.47 ± 4.36 b
	36 h	69.87 ± 0.86 a	68.99 ± 0.27 ab
	48 h	70.52 ± 0.78 a	69.90 ± 0.41 a
	60 h	69.56 ± 1.15 ab	70.16 ± 0.71 a
b*	0 h	34.23 ± 2.11 abc	33.20 ± 1.38 bcd
	8 h	35.32 ± 0.57 a	30.87 ± 0.86 ef
	16 h	34.46 ± 0.89 ab	32.80 ± 0.69 bcd
	24 h	33.01 ± 0.51 bcd	29.55 ± 2.24 fg
	36 h	31.54 ± 1.49 de	28.82 ± 0.85 g
	48 h	32.68 ± 0.85 bcde	29.31 ± 0.41 fg
	60 h	28.71 ± 0.98 g	32.47 ± 1.04 cde

Note: values are average values ± standard deviation (biological replicates, n = 3). Different lowercase letters (a, b, c, d, e, f and g) within the same row indicate significant differences over time within each treatment group (*p* < 0.05).

**Table 2:** Color changes in sweet corn during *F. verticillioides* infection.

Color	Storage Time	CK	FV
L*	0 h	69.80 ± 0.72 ab	68.05 ± 1.57 bc
	12 h	70.36 ± 1.09 a	68.62 ± 1.02 abc
	24 h	70.32 ± 0.16 a	69.38 ± 0.82 ab
	36 h	69.87 ± 0.86 ab	67.21 ± 1.17 c
	48 h	70.52 ± 0.78 a	69.03 ± 0.68 abc
	60 h	69.56 ± 1.15 ab	68.97 ± 1.81 abc
b*	0 h	34.23 ± 2.11 a	31.91 ± 2.75 abcd
	12 h	33.13 ± 2.05 abc	33.76 ± 0.73 ab
	24 h	33.01 ± 0.51 abc	32.37 ± 0.90 abc
	36 h	31.54 ± 1.49 abcd	29.70 ± 2.54 cd
	48 h	32.68 ± 0.85 abc	30.26 ± 0.98 bcd
	60 h	28.71 ± 0.98 d	29.59 ± 3.33 cd

Note: values are average values ± standard deviation (biological replicates, n = 3). Different lowercase letters (a, b, c and d) within the same row indicate significant differences over time within each treatment group (*p* < 0.05).

## Data Availability

The original contributions presented in the study are included in the article/Supplementary Materials; further inquiries can be directed to the corresponding author.

## Supplementary Materials

The following supporting information can be downloaded at https://www.mdpi.com/article/10.3390/foods14234147/s1, Table S1: Sweetcorn firmness detection parameters.
