# Supplementary material for: Effects of Fusarium graminearum and Fusarium verticillioides Infection on Sweet Corn Quality During Postharvest Storage
Source: Foods. 2025 Dec 3;14(23):4147. doi: 10.3390/foods14234147 (PMC12692720; doi:10.3390/foods14234147)
Supplement: Supplementary file 1 [file foods-14-04147-s001.zip › foods-3956673-supplementary.pdf]

Table S1. Sweetcorn firmness detection parameters.

Parameter Settings	
Pre-test Rate	1 mm/s
Test Rate	2 mm/s
Post-test Rate	2 mm/s
Target Mode	Compression
Compression Level	70%
Time	5 s
Trigger Type	Automatic (Force)
Trigger Force	3 N
